# Using Hepatitis B Surface Antigen–Positive Donors in Liver Transplantation: Outcomes, Prophylaxis, and Monitoring with a Focus on Hepatitis B Virus–Naive Recipients

**DOI:** 10.5152/tjg.2026.26018

**Published:** 2026-05-13

**Authors:** Ali Emre Bardak, Aafeen Mujeeb, Ayse Eylul Donmez, Nazli Begum Ozturk, Eda Yildiz, Hemeesh Tandel, Merih Deniz Toruner, Merve Gurakar, Behnam Saberi, Nilay Danis, Ahmet Gurakar

**Affiliations:** 1 Department of Internal Medicine, Boston Medical Center, Boston, MA, USA; 2 Division of Gastroenterology and Hepatology, Johns Hopkins University School of Medicine, Baltimore, MD, USA; 3 Children’s Hospital, University of Illinois, Chicago, USA; 4 Division of Gastroenterology and Hepatology, Saint Louis University School of Medicine, St. Louis, MO, USA; 5 Warren Alpert Medical School of Brown University, RI, USA; 6 Division of Gastroenterology and Hepatology, Beth Israel Deaconess Medical Center, Harvard Medical School, Boston, MA, USA; 7 Division of Gastroenterology, Department of Internal Medicine, Dokuz Eylul University, İzmir, Türkiye

**Keywords:** Donors and donation, liver transplantation, viral hepatitis B

## Abstract

Liver transplantation (LT) remains the only definitive treatment for end-stage liver disease and selected hepatic malignancies. However, persistent organ shortages continue to drive high waitlist mortality worldwide. Hepatitis B virus (HBV)-positive donors, including hepatitis B surface antigen (HBsAg)-positive and anti-hepatitis B core antibody–positive (HBsAg-negative) donors, represent an increasingly important but historically underused resource. The introduction of high-barrier nucleos(t)ide analogs, the selective use of hepatitis B immunoglobulin, and pre- and posttransplant vaccination have markedly reduced viral transmission, enabling safe transplantation in both HBV-immune and selected HBV-naive recipients. Regional guidelines and consensus statements, including those from the American Society of Transplantation, European Association for the Study of the Liver, American Association for the Study of Liver Diseases, and Asian Pacific Association for the Study of the Liver, support individualized, risk-directed prophylaxis and posttransplant surveillance. Ethical considerations, particularly informed consent and risk–benefit discussions in the setting of organ scarcity, remain central to clinical decision-making. Large cohort studies demonstrate comparable graft and patient survival with HBV-negative grafts, along with economic advantages and expansion of the donor pool. However, evidence in HBV-naive recipients remains limited, with most data derived from immune or previously infected recipients, highlighting the need for careful patient selection and robust prophylaxis. Available evidence on the use of HBsAg-positive donors in HBV-naive recipients is obtained predominantly from cadaveric donor transplantation, as experience with HBsAg-positive living donors remains limited. This narrative review summarizes current evidence on the use of HBsAg-positive grafts in LT, with a focus on HBV-naive recipients, based predominantly on cadaveric donor experience.

**Main Points**

- HBV-positive donors can be categorized into three clinically relevant groups: HBsAg-positive/HBV-DNA-positive donors, HBsAg-positive/HBV-DNA-negative donors, and anti-HBc-positive (HBsAg-negative) donors.
- HBsAg-positive/HBV-DNA-positive grafts carry the highest risk of viral transmission; HBsAg-positive/HBV-DNA-negative grafts also carry substantial risk; and anti-HBc-positive (HBsAg-negative) grafts, when managed carefully, carry a lower but still non-negligible risk.
- Although de novo Hepatitis B infection (DNH) development is a major risk in recipients, evidence increasingly shows that antiviral prophylaxis strategies can significantly reduce DNH risk and findings are comparable with HBV negative donors, suggesting carefully selected HBV-naïve individuals benefit from HBV positive grafts by the protective effect of prophylaxis.
- The potential benefits of using safe HBV-positive grafts include reducing waitlist mortality, expanding transplant options, and being cost-effective.
- Limited long-term data in HBV-naïve recipients, the predominance of cadaveric donor–derived evidence for HBsAg-positive graft use, and the lack of standardized global protocols can be considered as remaining significant limitations.

## Introduction

Liver transplantation (LT) remains the gold standard for treating end-stage liver disease and certain liver cancers. However, the severe shortage of donor organs remains a global problem, and the persistent imbalance between organ supply and demand limits transplantation programs worldwide, resulting in thousands of deaths each year among patients awaiting transplant. Although the number of liver transplants in the United States increased by 7.5% in 2024 compared with 2023, the organ shortage persists; as of September 2024, 9424 people remained on the waiting list.^1^^,^^2^ To address this issue, transplant centers are increasingly exploring strategies to expand the donor pool, and one promising option is using grafts from hepatitis B virus (HBV)–positive donors, which were once considered unsuitable.

HBV-positive donors are classified into 3 categories: hepatitis B surface antigen (HBsAg) positive/HBV DNA positive, indicating active infection and viral replication; HBsAg positive/HBV DNA negative, indicating active infection; and hepatitis B core antibody (anti-HBc) positive (HBsAg negative), indicating prior exposure. HBsAg-positive grafts carry the highest risk of viral transmission. In contrast, anti-HBc-positive (HBsAg-negative) grafts pose a lower and more variable risk.^3^ Historically, organs from these donors were avoided because of high rates of recurrent infection. However, the introduction of potent antivirals, the refined use of hepatitis B immune globulin (HBIG), widespread vaccination, and improved selection strategies have markedly altered this risk profile.^4^^,^^5^

Evidence from multiple studies indicates that anti-HBc-positive (HBsAg-negative) grafts achieve favorable results, particularly in immune recipients (anti-HBs positive). Even seronegative recipients (anti-HBc negative, HBsAg negative, and anti-HBs negative) have favorable outcomes with appropriate prophylaxis.^6^^,^^7^ Although data on HBsAg-positive donors remain relatively limited, growing experience with this group, especially from Asian centers, suggests these grafts can be used selectively with close monitoring.^8^^,^^9^ Importantly, their use is not only clinically viable but also economically advantageous, reducing waitlist mortality and health care costs.^10^ Therefore, consensus guidelines increasingly recommend the use of HBsAg-positive grafts within defined protocols.^11^

## Materials and Methods

This narrative review summarizes current evidence on outcomes, prophylaxis strategies, special populations, economic impact, and evolving guideline perspectives related to the safe use of HBV-positive donors in LT, with particular emphasis on HBV-naive recipients (anti-HBc negative and HBsAg negative) and individualized donor–recipient matching, highlighting that most available data are obtained from deceased donor transplantation and that data from living donor transplantation remain limited. This narrative synthesis was conducted by searching literature from PubMed/MEDLINE, Embase, and Google Scholar for English-language studies published between 2000 and 2025, using terms related to LT, HBsAg-positive donors, and donor and recipient HBV serology. Titles and abstracts were screened, followed by full-text review of eligible articles. Additional relevant studies were identified through manual review of reference lists of included articles.

### Hepatitis B Virus–Positive Donor Classification and Utilization in Liver Transplantation

HBV-positive donors can be broadly classified as HBsAg-positive donors and anti-HBc-positive (HBsAg-negative) donors. Among HBsAg-positive donors, HBV DNA status further stratifies risk into HBsAg-positive/HBV DNA-positive (hereafter HBV DNA-positive) and HBsAg-positive/HBV DNA-negative donors. Each group has distinct virologic features, risks, and clinical implications (Table 1, Figure 1).^8^^,^^11^^,^^12^ Transplant medicine no longer categorizes HBV-positive donors as a universally high-risk cohort. Appropriate use of these donor grafts depends heavily on thorough serologic assessment and careful recipient matching. In the early LT era, the use of HBV-positive donor livers elicited substantial hesitation because of the high risk of viral transmission and subsequent graft failure. However, with the emergence of sensitive diagnostic tests, potent antiviral prophylaxis, and improved understanding of immune-mediated defense mechanisms, clinical attitudes toward HBV-positive grafts have shifted considerably toward selective use.^12^^-^^14^

HBV DNA-positive donors are recognized as a distinct high-risk subgroup, necessitating more stringent prophylaxis and post-LT monitoring.^14^^,^^15^ Historically, even HBsAg-positive/HBV DNA-negative donors were considered unsuitable for LT because of the high risk of viral transmission driven by the persistence of intrahepatic covalently closed circular DNA (cccDNA) and residual HBV DNA. However, data from the past 2 decades support their use in selected cases, with several studies demonstrating comparable long-term survival rates with careful prophylaxis and monitoring. For instance, in a long-term follow-up series of 28 LT recipients from HBsAg-positive donors, 75% were HBV DNA positive and 25% HBV DNA negative. The 1-, 3-, and 5-year graft survival rates were 85.7%, 82.1%, and 78.4%, respectively; outcomes were reported for the overall cohort without stratification by donor HBV DNA status.^16^ A recent study compared the survival rates between HBsAg-positive (not stratified by HBV DNA status) and HBsAg-negative graft recipients who were HBsAg negative before LT. Analyses showed no statistically significant difference between the 2 groups.^17^ An analysis of the United Network for Organ Sharing (UNOS) registry from 2009 to 2020 identified 70 LT using HBsAg-positive donors (~0.7% of donors in that period). Most of these grafts (58 of 70) were transplanted into patients without chronic HBV infection. The data were encouraging: the 1- and 3-year posttransplant survival rates were 96.6% and 91.4%, respectively, comparable to those of propensity-matched HBsAg-negative grafts.^11^ Importantly, this UNOS registry analysis did not stratify HBsAg-positive donors by HBV DNA status because donor viremia data were unavailable; outcomes were reported for the overall HBsAg-positive donor cohort. Despite these favorable outcomes, a recent international survey of 135 LT centers found that HBsAg-positive donors remain underutilized worldwide.^18^ Only 40% of centers reported national or regional approval for their use, with an additional 20% permitting their use under special circumstances. Notably, centers with higher waitlist mortality were more likely to use these grafts. Similarly, in endemic regions such as Asia, where HBV prevalence is higher, clinicians adopt a more flexible approach, particularly when donor viral load is low or suppressed.^19^

Anti-HBc-positive (HBsAg-negative) donors indicate past exposure or resolved infection and are more common than HBV-positive donors. Viral replication rate in these donors is typically low or absent; therefore, they pose a lower, though not negligible, risk of transmission. The risk of HBV transmission depends largely on the donor HBV DNA status, the recipient immune status, and the use of antiviral prophylaxis.^3^^,^^20^ In modern practice, these grafts are widely accepted, particularly when recipients are immune or vaccinated and appropriate prophylaxis is used.^3^^,^^12^^,^^20^

Overall, HBV DNA-positive grafts carry the highest risk of viral transmission. HBsAg-positive/HBV DNA-negative grafts carry a lower but still considerable risk of viral transmission, necessitating careful recipient selection, optimized antiviral prophylaxis, and preferential allocation to recipients with HBV infection or immunity. In contrast, anti-HBc-positive grafts, when managed appropriately, carry a lower risk and are now widely used in LT worldwide.^3^^,^^8^^,^^14^^,^^20^

### Risk of De Novo Hepatitis B

The main concern following the use of HBV-positive donor livers in HBV-naive recipients is the development of de novo hepatitis B (DNH), defined as the posttransplant appearance of HBV infection in recipients who were initially HBV-naive. It typically occurs after transplantation of a graft from an HBV-positive donor.^7^^,^^12^ Without prophylaxis, the risk of DNH in recipients of anti-HBc-positive (HBsAg-negative) grafts is 30%-60%, whereas it approaches 100% in seronegative recipients of HBsAg-positive grafts because of persistent intrahepatic cccDNA and residual HBV DNA.^3^^,^^12^^,21^ Historically, DNH has been associated with a substantial risk of chronic hepatitis, progressive graft dysfunction, and ultimately graft failure. Accordingly, most centers refrained from using anti-HBc-positive or HBsAg-positive grafts, which posed a major barrier to expansion of the donor pool. With the introduction of potent nucleos(t)ide analogs (NAs) and tailored prophylaxis strategies, the risk of DNH has become more controllable and relatively less worrisome (Figure 2).

In a 2020 retrospective study of 19 adult living-donor liver transplant recipients of anti-HBc-positive grafts, 7 patients discontinued prophylaxis 8-24 months posttransplant; 6 of these subsequently developed DNH. In contrast, among the 12 patients who continued lamivudine and HBIG, only 1 developed DNH, highlighting the importance of sustained antiviral prophylaxis in minimizing the risk of DNH.^22^

A systematic review of 39 studies including 903 recipients reported that among HBsAg-negative transplant patients receiving anti-HBc-positive (HBsAg negative) grafts, DNH occurred in nearly half (48%) of those without prophylaxis. In contrast, recipients with pre-existing immunity (anti-HBc- or anti-HBs-positive) had a markedly lower infection rate of approximately 15%.^3^ This demonstrates that recipient immune status at the time of LT influences the incidence of DNH. Another systematic review of 26 studies including 462 anti-HBc-positive (HBsAg-negative) graft recipients reported that the risk of DNH was highest among HBV-naive recipients who received no prophylaxis, reaching 58%. In this group, antiviral prophylaxis (lamivudine and/or HBIG) reduced the incidence of DNH to 11%. Among anti-HBs-positive and anti-HBc-negative recipients, DNH occurred in 18% without prophylaxis and 0% with prophylaxis, whereas recipients who were both anti-HBs positive and anti-HBc positive had DNH rates of 4% without prophylaxis and 3% with prophylaxis. Recipients who were anti-HBc positive had DNH rates of 14% without prophylaxis and 3% with prophylaxis. Overall, these findings demonstrate that antiviral prophylaxis significantly reduces the risk of DNH, with the greatest benefit observed among HBV-naive individuals, highlighting the added protective value of pre-existing immune memory.^7^

Consistent observations have been reported in the pediatric population. In a pediatric cohort of 482 LT recipients, including 170 recipients of anti-HBc-positive grafts,^23^ DNH occurred in 11 recipients, 10 of whom had received anti-HBc-positive grafts. The incidence of DNH was also assessed according to pre- and post-LT anti-HBs levels. Patients meeting both pretransplant anti-HBs ≥1000 IU/L and posttransplant anti-HBs ≥200 IU/L thresholds developed significantly lower DNH (1.3%) than those meeting only 1 criterion (4.2% and 1.9%, respectively). De novo hepatitis B incidence was 43.8% when neither criterion was met. Similarly, a randomized controlled trial in children undergoing LT with waning HBV immunity showed that a structured booster vaccination schedule effectively restored and maintained protective anti-HBs levels over time. This reinforces the importance of planned revaccination and serial antibody monitoring to preserve long-term protection against DNH in this population.^24^ These results demonstrate the importance of recipient immune status, both before and after LT, in reducing the risk of DNH.

### Prophylactic Strategies

As described earlier, the risk of DNH is very high in HBV-naive recipients of HBsAg-positive or anti-HBc-positive grafts without prophylaxis. However, with proper antiviral prophylaxis, long-term graft durability has significantly improved, and anti-HBc- and even HBsAg-positive grafts, once considered unacceptably high risk, can now serve as valuable and potentially lifesaving organs.^14^ High-barrier NAs, selective use of HBIG, and targeted vaccination are the 3 main components of contemporary antiviral prophylaxis. High-barrier NAs are considered first-line agents. HBIG is now used more selectively, generally in higher-risk settings such as HBsAg-positive grafts, to provide immediate passive immunity and reduce early posttransplant viral rebound. Vaccination remains an essential adjunct for HBV-naive or nonimmune recipients. Successful immunization, particularly when protective anti-HBs titers are achieved pretransplant and maintained posttransplant, offers durable immune protection and may allow for simplified prophylactic regimens.

High-barrier NAs, including entecavir, tenofovir disoproxil fumarate (TDF), and tenofovir alafenamide (TAF), have transformed HBV prophylaxis in LT. In HBV-naive recipients of HBsAg-positive or anti-HBc-positive grafts, one of these agents should be initiated as early as possible after LT and continued indefinitely, regardless of recipient HBV DNA status.^12^^,^^14^ These drugs combine high potency with very low resistance rates (<1% in treatment-naive patients) and favorable safety profiles. Dosing is adjusted based on renal function, age, and prior lamivudine exposure or resistance (e.g., a higher entecavir dose for lamivudine resistance).^14^^,^^25^ Patients who develop renal impairment or bone disease while receiving TDF are typically switched to TAF or entecavir.^26^^,^^27^ A systematic review of 17 studies including 519 chronic HBV LT recipients found that high-genetic-barrier NA therapy (entecavir or TDF), with or without HBIG, substantially reduced HBV recurrence compared with lamivudine plus HBIG (1.0% vs. 6.1%), Recurrence remained low after HBIG discontinuation.^28^ In a cohort of 964 LT recipients, outcomes were comparable between recipients of isolated anti-HBc-positive (HBsAg-negative) donors and those receiving anti-HBc–negative donors.^20^ A total of 108 HBsAg-negative recipients of anti-HBc-positive (HBsAg negative) grafts received NA monotherapy. De novo hepatitis B occurred in only 2.8%, all of whom were receiving lamivudine; none were receiving entecavir. Regarding long-term outcomes, a series of 265 recipients with chronic HBV LT managed with entecavir monotherapy demonstrated durable virologic control (HBV DNA undetectable in 95%-100% through 8 years), HBsAg seroclearance in 92%, and 9-year survival of 85%. None of the deaths were attributable to HBV recurrence.^29^

HBIG still has a role, but its use is now individualized and risk-directed rather than routine. The current approach reserves HBIG, always in combination with a high-barrier NA when used, for higher-risk scenarios, such as HBV-positive grafts with absent or insufficient recipient anti-HBs or HBV/HDV (Hepatitis B Virus/Hepatitis D Virus) coinfection. In lower-risk settings, monotherapy with a high-barrier NA is used.^30^^,^^31^ A meta-analysis of 51 studies showed that combination therapy with HBIG and NA reduced recurrence more than NA monotherapy (OR = 0.22; 95% CI: 0.16-0.30). However, many NA monotherapy comparators used lamivudine, a low-barrier NA. In subgroup analysis comparing combination therapy with HBIG and high-barrier NA (entecavir or tenofovir) versus high-barrier NA monotherapy, no significant difference was observed (OR = 0.70; 95% CI: 0.22-2.26).^32^ Similarly, another meta-analysis of 19 studies showed that HBIG did not provide additional benefit in HBV DNA–negative recipients or when high-barrier NAs were used.^33^ The apparent benefit of adding HBIG is therefore driven mainly by lamivudine-era comparators; with modern high-barrier NA therapy, no significant benefit of HBIG has been demonstrated.

In a single-center risk-directed protocol of 20 HBsAg-positive recipients, low-risk patients (Hepatitis B e antigen [HBeAg] negative, HBV DNA < 12 IU/mL) received only a very short HBIG course (median 7 days) before transitioning to entecavir or TDF monotherapy, whereas high-risk patients continued HBIG ≥ 6 months. Over approximately 4 years of follow-up, there was no HBV DNA recurrence or clinically significant hepatitis, and graft function remained stable. Two patients had isolated, low-level HBsAg reappearance without viremia; both had pre-LT hepatocellular carcinoma (HCC), suggesting HBsAg expression from integrated DNA rather than true reinfection.^34^

Vaccination complements NA-based prophylaxis and, when feasible, should be administered before LT, targeting an anti-HBs level of ≥1000 IU/L pretransplant and maintaining ≥100 IU/L posttransplant, with booster doses as needed. Post-LT vaccination alongside NA therapy remains worthwhile. Although immune responses may be blunted, anti-HBs titers still increase, helping to maintain protective immunity.^12^^,^^35^^,^^36^ In pediatrics, a combination of NA and active immunization has reduced DNH to near zero over multiyear follow-up. In adults, pre- or post-LT vaccination increases anti-HBs levels and reduces the risk of DNH, even in seronegative LT recipients.^23^^,^^37^

HBV prophylaxis requires continuous attention rather than a “set-and-forget” approach. Routine posttransplant surveillance for HBsAg and HBV DNA is essential to detect early viral reactivation or occult infection and to allow timely adjustment of therapy. If HBV DNA becomes detectable, therapy can be intensified by adding or reinitiating HBIG and optimizing NA therapy.^14^^,^^15^

### Clinical Outcomes with Hepatitis B Surface Antigen–Positive Grafts

Without prophylaxis, HBV transmission from an HBsAg-positive donor to an HBV-naive recipient is nearly universal, and mortality approaches 50% within 2 years because of fulminant hepatitis or graft failure.^9^^,^^14^^,^^38^ In practice, allocation policies preferentially place HBsAg-positive grafts in immune or already HBsAg-positive recipients to avoid introducing a new infection. Consequently, HBV-naive recipients of HBsAg-positive grafts are uncommon, and the evidence base in this group remains limited.^9^^,^^39^ Even so, given the organ shortage, some centers offer HBsAg-positive livers to HBV-naive candidates who are critically ill and unlikely to survive the wait for transplantation. When recipients provide informed consent and have no contraindications to lifelong antivirals, the decision helps to strike a balance between the immediate lifesaving measures and the risk of long-term infection that can be more manageable.^11^^,^^40^ A cost-effectiveness analysis demonstrated that accepting an HBsAg-positive liver for an HBV-naive recipient is cost-effective, particularly at higher Model for End-Stage Liver Disease (MELD) scores, supporting that with modern antiviral prophylaxis, the use of HBsAg-positive grafts is both clinically defensible and economically reasonable and may help expand the donor pool without excessive cost.^10^

In the United States, utilization of HBsAg-positive donor livers increased from 6 to 19 grafts per year between 2009 and 2020, although more than half of the offered HBsAg-positive livers were still discarded in 2020. During this period, 70 recipients received these grafts; the mean age was 56.2 years, 61.4% were male, and the mean MELD score was 20.7. Most recipients (58/70; 82.9%) were HBV naive. Among HBV-naive recipients with available serology, 17 (approximately 41%) had anti-HBs positivity. Post-LT survival was considered promising, with 1- and 3-year survival rates of 95.7% and 91.4%, respectively. For HBV-naive recipients specifically, 1- and 3-year survival rates were 96.6% and 91.4%, respectively, with no statistically significant difference compared with HBV-positive recipients. The observed survival rates are comparable to national adult LT outcomes with HBsAg-negative donors.^11^ Complementing these population-level data, a single-center series of 10 HBsAg-negative recipients of HBsAg-positive grafts between 2019 and 2021 demonstrated comparable survival rates over approximately 4 years of follow-up to other transplants performed during the same period. Most patients maintained undetectable HBV DNA, and approximately one-third cleared HBsAg during follow-up.^17^ In a larger single-center cohort including 42 non-HBV-naive recipients of HBsAg-positive grafts and 327 recipients of HBsAg-negative grafts, HBsAg-positive graft use under NA prophylaxis resulted in survival and complication rates comparable to standard grafts. The study also evaluated the impact of HBIG on early HBsAg and HBV DNA clearance and concluded that, although HBIG may partially neutralize HBsAg, it does not contribute to virologic clearance. Prophylaxis with NA monotherapy appears safe and sufficient.^8^ An Italian multicenter experience from 2004 to 2010 further supports feasibility under prophylaxis: among 28 recipients of HBsAg-positive grafts, 5 were HBsAg negative but had evidence of prior HBV infection rather than true naive status. All patients received lamivudine with HBIG, with HBIG dosing adjusted to maintain anti-HBs levels of >250 IU/mL. Patient and graft survival rates at 1, 3, and 5 years were 85.7%, 82.1%, and 78.4%, respectively. Four patients had HBV recurrences, none of which compromised survival.^16^ A Taiwanese center reported data on 14 LT from HBsAg-positive donors who were HBeAg-negative with undetectable HBV DNA. Prophylaxis was managed with entecavir monotherapy without the use of HBIG. One-, 2-, and 4-year survival rates were 100%, 92.9%, and 77.4%, respectively, and were comparable with those after LT from HBsAg-negative donors. During follow-up, HBV DNA remained undetectable, and no recurrence was detected. However, it should be noted that the cohort included no truly HBV-naive recipients; all but 1 were HBsAg-positive at baseline, and the single HBsAg-negative case was anti-HBc positive.^19^ Similarly, a multicenter Chinese cohort consisting mostly of non-naive, HBV-related indications showed comparable early and late complications and 1-, 3-, and 5-year survival rates between recipients of HBsAg-positive and HBsAg-negative grafts. However, HBsAg-positive grafts were associated with higher HBV recurrence, likely reflecting the predominant use of low-barrier NAs with limited HBIG rather than modern high-barrier NA-based strategies with or without HBIG.^41^ Although data remain limited, the use of HBsAg-positive grafts in HBV-naive patients is considered safe and feasible; however, existing safety data are strongest in non-naive populations, and evidence in truly naive recipients remains limited.

When HCC is the indication for LT, oncologic outcomes can outweigh virologic considerations and significantly influence overall survival. A propensity-matched Chinese HCC cohort consisting entirely of HBsAg-positive recipients showed that HBsAg-positive donor grafts had similar perioperative and early graft outcomes under HBIG plus entecavir prophylaxis. However, HBsAg-positive grafts were associated with worse 1- and 3-year progression-free and overall survival rates, with donor HBsAg positivity identified as an independent risk factor. These findings appeared to be driven primarily by HCC recurrence rather than HBV reinfection.^42^ HBsAg plays a crucial role in the occurrence, progression, and postoperative recurrence of HBV-associated HCC, affecting various cellular processes such as proliferation, endoplasmic reticulum (ER) stress, cell cycle regulation, metabolic reprogramming, and immune suppression.^43^ These findings support stricter recipient selection and enhanced oncologic surveillance when considering HBsAg-positive grafts for HCC.

Beyond HBsAg-positive donors, experience with donors with detectable HBV DNA also demonstrates strong virologic control under prophylaxis. In a single-center cohort of 144 LT recipients from anti-HBc-positive grafts, 57 donor livers had detectable HBV DNA by nucleic acid testing. Prophylaxis consisted of indefinite NA therapy, with a single intraoperative HBIG dose administered only to vaccine nonresponders; recipients of grafts from donors without detectable HBV DNA did not receive HBIG. Post-LT viremia occurred more frequently and earlier in donors with detectable HBV DNA (14.8% vs. 3.5%; median 1.2 vs. 9.0 months). The presence of detectable HBV DNA independently predicted posttransplant viremia. Nevertheless, all recipients in the detectable-HBV DNA group achieved HBV DNA negativity on therapy by the last follow-up at a median of 36 months, and patient and graft survival were comparable with those in recipients without detectable donor HBV DNA.^44^

Taken together, these data support risk-directed expansion of the donor pool with HBsAg-positive and detectable HBV DNA grafts, using high-barrier NAs and selective HBIG in carefully selected patients. Most published experience with HBsAg-positive grafts reflects deceased donor transplantation, whereas dedicated data on HBsAg-positive living donors are sparse. As a result, donor-type–specific risk estimates and strategies for living donation are less well defined, and recommendations are often extrapolated from deceased donor experience. In addition, most evidence derives from non-HBV-naive recipients; therefore, special consideration is warranted for truly HBV-naive patients.

### Guidelines and Consensus Statements

Over the past decade, policies regarding the use of HBsAg-positive donors in LT have evolved from total exclusion to a more evidence-based, risk-directed framework. Current guidelines emphasize individualized assessment, effective antiviral protection, and close post-LT monitoring rather than blanket exclusion (Figure 3).

The 2015 consensus statement from the American Society of Transplantation (AST) laid the groundwork for this approach, endorsing the consideration of HBsAg-positive or anti-HBc-positive donors under structured protocols, along with extended NA therapy for recipients at risk.^12^ The guidance remains pivotal, with later global recommendations building upon it. The 2025 Clinical Practice Guidelines from the European Association for the Study of the Liver (EASL) reaffirm and further refine this strategy.^45^ According to EASL, HBsAg-positive grafts may be used in selected patients under specific conditions, including those without prior immunity, given that a high-barrier NA therapy is initiated around the time of LT and continued long term; HBV DNA and HBsAg levels are closely monitored over time; and the LT is performed at centers with extensive experience and established antiviral protocols. Importantly, the newly published 2025 American Association for the Study of Liver Diseases/American Society of Transplantation Practice Guideline (AASLD/AST) practice guideline explicitly addresses HBsAg-positive graft use in recipients without HBV infection.^46^ The guideline states that HBsAg-positive grafts may be considered in HBV-negative adult LT recipients with first-line NA therapy to suppress viremia, with or without HBIG, after a thorough multidisciplinary discussion of risks and benefits between the candidate and transplant team This recommendation is based on limited, predominantly observational evidence rather than large randomized trials and therefore requires individualization. In the Asia-Pacific region, which has the highest HBV burden, the 2015 Asian Pasific Association for the Study of the Liver (APASL) recommendations similarly endorsed the use of carefully selected HBsAg-positive and anti-HBc-positive grafts, provided effective prophylactic treatment is in place.^47^ These principles remain applicable and have guided more permissive transplantation practices in endemic countries, where the shortage of suitable donors poses a major challenge. Beyond society guidelines, a 2024 International Liver Transplantation Society(ILTS)–EASL–AASLD multisociety survey found that nearly 60% of LT centers globally now accept the use of HBsAg-positive grafts under established protocols, although practice heterogeneity persists.^18^ Survey evidence suggests that real-world adoption is increasing in parallel with evolving guidelines.

Overall, contemporary guidelines recommend the careful use of HBsAg-positive donor livers for HBV-naive recipients, provided that potent antiviral therapy, personalized risk assessment, and vigilant follow-up are implemented. Together, these recommendations represent a pivotal step toward safely broadening the donor pool.

### Ethical and Clinical Considerations

Ethical considerations are fundamental when using HBV-positive donors in LT. Recipients, particularly HBV-naive recipients, should be informed of possible risks and benefits, such as the risk of DNH, the manageability of these risks, the prophylactic value of high-barrier antiviral agents, and the long-term monitoring requirements. Higher-risk grafts are preferably allocated to recipients who are HBV positive or have evidence of prior immunity to HBV (anti-HBs or anti-HBc positivity).

Benefits should exceed the risks when such grafts are considered. The potential benefits of HBV-positive grafts include reduced waitlist mortality, expanded access to transplantation, and cost-effectiveness.

Transplant teams can enhance access while maintaining safety by adopting guideline-based strategies combined with patient-centered, ethical decision-making. This approach enables safe expansion of the donor pool while preserving trust and optimizing patient outcomes.

The growing body of evidence has shifted the use of HBsAg-positive liver grafts from a previously prohibitive option to a viable and ethically supportable strategy in carefully selected settings. With potent antiviral prophylaxis, appropriate recipient selection, and structured post-LT monitoring, graft and patient outcomes can be comparable with those achieved using HBsAg-negative donors, expanding the donor pool without compromising safety.

Nonetheless, significant limitations remain, including the limited and largely observational nature of the available data in HBV-naive recipients, a predominance of cadaveric donor–derived evidence for HBsAg-positive graft use (with limited living-donor experience), and the absence of standardized global protocols. A limitation is that outcomes are often not stratified by donor HBV DNA status, which further restricts donor-type–specific recommendations. Future studies should aim to streamline prophylaxis, improve long-term graft and patient survival in heterogeneous populations, develop donor-type–specific recommendations, and standardize regional guidance to ensure equitable and safe use of HBV-positive organs.

Ultimately, the use of HBV-positive liver grafts may represent a promising strategy for expanding the donor pool when carefully implemented with evidence-based strategies and appropriate patient selection. This approach may help address organ scarcity and serve as a model for ethically grounded, data-driven innovative approaches in transplantation medicine.

## Figures and Tables

**Figure 1.**
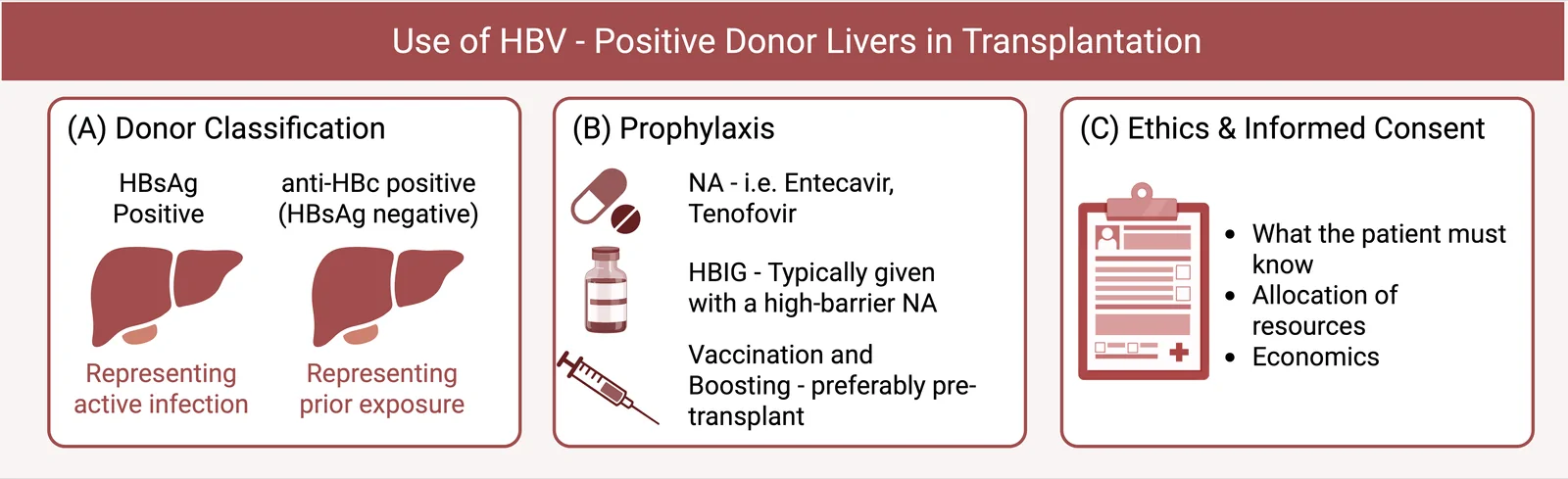
Overview of the use of HBV-positive donor livers in liver transplantation. (A) Donor classification, distinguishing HBsAg-positive donors (active infection) from anti-HBc-positive (HBsAg negative) donors (prior exposure). (B) Prophylaxis strategies, including NA, use of HBIG, and vaccination. (C) Ethical considerations, including informed consent and economic implications. HBc, hepatitis B core; HBIG, hepatitis B immunoglobulin; HBsAg, hepatitis B surface antigen; HBV, hepatitis B virus; NA nucleos(t)ide analogs.

**Figure 2.**
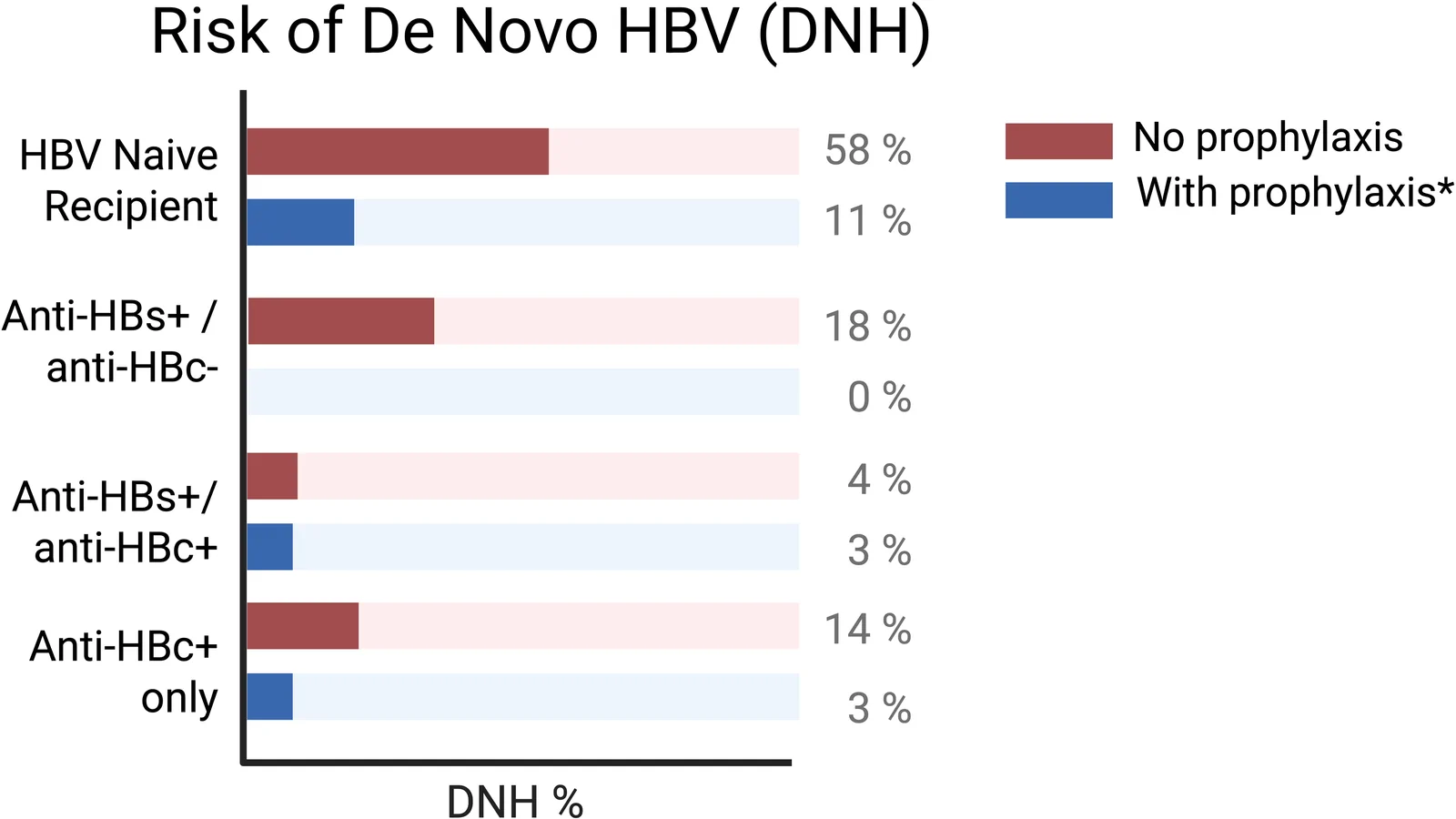
Risk of DNH after transplantation using anti-HBc-positive (HBsAg-negative) donor livers. Risk of DNH is depicted here by the recipient’s immune status with and without prophylaxis. Data derived from Skagen et al^7^. DNH, de novo hepatitis B; HBc, hepatitis B core; HBsAg, hepatitis B surface antigen; HBV, hepatitis B virus.

**Figure 3.**
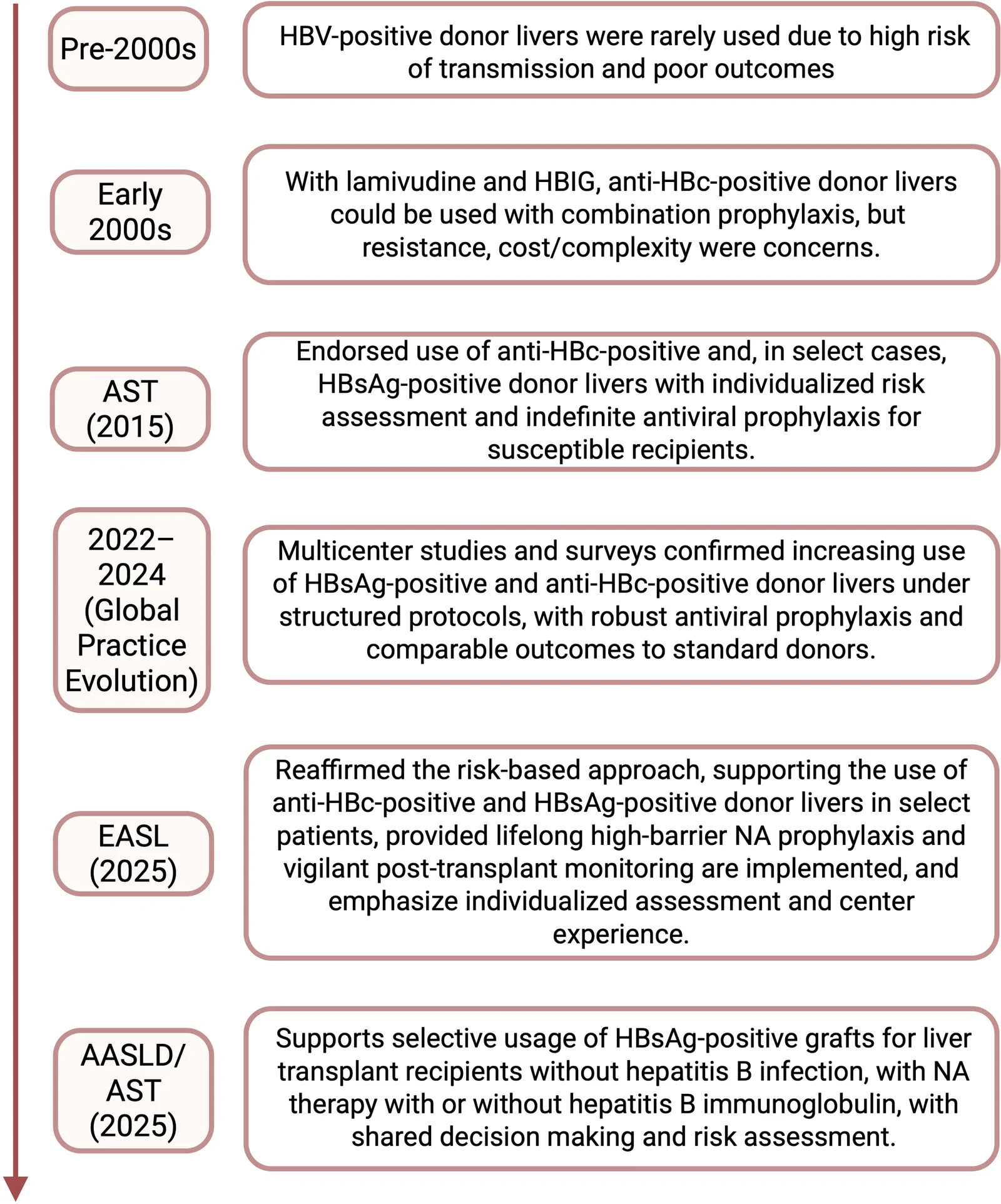
Evolution of clinical guidelines for the use of HBV-positive donor livers in transplantation. Depiction of changes in guidelines regarding HBV-positive donor livers in transplantation. Consensus statements and guidelines from AST (2015),^12^ APASL (2015),^46^ EASL (2025),^44^ and AASLD/AST (2025)^45^ progressively endorsed a risk-stratified approach, emphasizing NA therapy, selective HBIG use, and close monitoring. AST, American Society of Transplantation; EASL, European Association for the Study of the Liver; HBIG, hepatitis B immunoglobulin; NA, nucleos(t)ide analog.

**Table 1.** Summary of Hepatitis B Virus–Positive Donor Types and Recipient De Novo Hepatitis B Risk

**Donor Category**	**Recipient-Related Risk (Highest → Lowest)**	**DNH/ Transmission Risk **	**Practical Prevention**	**Monitoring and Key Nuances**
HBsAg+/HBV DNA+	HBV-naive/nonimmune (anti-HBs−) → HBV-immune (anti-HBs+) → prior exposure (anti-HBs+/anti-HBc+)	Highest-risk subgroup; DNH risk is very high in HBV-naive without prophylaxis	High-barrier NA indefinitely; consider HBIG in higher-risk settings (e.g., anti-HBs absent/low), acknowledging uncertainty on incremental benefit with modern NA	Monitor HBsAg and HBV DNA. If breakthrough viremia occurs, optimize NA adherence/choice and consider HBIG per protocol
HBsAg+/HBV DNA−	HBV-naive/nonimmune → HBV-immune → prior exposure	Still high in HBV naive without prophylaxis; outcomes often reported for HBsAg+ donors without HBV DNA stratification	High-barrier NA indefinitely; HBIG individualized; vaccination/anti-HBs optimization as adjunct	Monitor HBsAg and HBV DNA. Treat DNA positivity (if later discovered) as a “risk escalator” → intensify monitoring/consider HBIG
Anti-HBc+ (HBsAg−)	HBV-naive/nonimmune → HBV-immune → prior exposure	Strongest quantitative DNH data; prophylaxis effects depend heavily on recipient baseline immunity. De novo hepatitis B ~50% without prophylaxis; markedly reduced with prophylaxis and pre-existing immunity^3^^,^^7^	NA prophylaxis lowers DNH. Low DNH with NA monotherapy; events mainly with lamivudine; none with entecavir^20^^,^^29^. Vaccination/anti-HBs maintenance adjunct	Monitor HBsAg and HBV DNA. Recipient anti-HBs/anti-HBc positivity meaningfully lowers risk; avoid lamivudine when possible (resistance concern)^20^^,^^29^

Outcomes for HBsAg-positive grafts are often reported without donor HBV DNA stratification; available evidence is predominantly from deceased donors, and data from living HBsAg-positive donors are limited.

Anti-HBc, hepatitis B core antibody; Anti-HBs, hepatitis B surface antibody; DNH, de novo hepatitis B; HBIG, hepatitis B immune globulin; HBsAg, hepatitis B surface antigen; HBV, hepatitis B virus; NA, nucleos(t)ide analog.

## Data Availability

The data that support the findings of this study are available on request from the corresponding author.
